# Outbreak with clonally related isolates of *Corynebacterium ulcerans* in a group of water rats

**DOI:** 10.1186/s12866-015-0384-x

**Published:** 2015-02-21

**Authors:** Tobias Eisenberg, Norman Mauder, Matthias Contzen, Jörg Rau, Christa Ewers, Karen Schlez, Gisa Althoff, Nicole Schauerte, Christina Geiger, Gabriele Margos, Regina Konrad, Andreas Sing

**Affiliations:** Landesbetrieb Hessisches Landeslabor, Schubertstr. 60, 35392 Gießen, Germany; Chemisches und Veterinäruntersuchungsamt Stuttgart, Schaflandstr. 3/2, 70736 Fellbach, Germany; Institute of Hygiene and Infectious Diseases of Animals, Justus Liebig University Giessen, Frankfurter Strasse 85-89, 35392 Giessen, Germany; Zoo Frankfurt, Bernhard-Grzimek-Allee 1, 60316 Frankfurt, Germany; National Consiliary Laboratory on Diphtheria, Bayerisches Landesamt für Gesundheit und Lebensmittelsicherheit, Veterinärstraße 2, 85764 Oberschleißheim, Germany

**Keywords:** *Corynebacterium ulcerans*, Water rat, *Hydromys chrysogaster*, Diphtheria toxin, FT-IR, MALDI-TOF MS, *rpoB*, *tox*, MLST, Persistent infection

## Abstract

**Background:**

The zoonotic bacterium *Corynebacterium ulcerans* may be pathogenic both in humans and animals: toxigenic strains can cause diphtheria or diphtheria-like disease in humans via diphtheria toxin, while strains producing the dermonecrotic exotoxin phospholipase D may lead to caseous lymphadenitis primarily in wild animals. Diphtheria toxin-positive *Corynebacterium ulcerans* strains have been isolated mainly from cattle, dogs and cats.

**Results:**

Here, we report a series of ten isolations of *Corynebacterium ulcerans* from a group of water rats (*Hydromys chrysogaster*) with ulcerative skin lesions, which were kept in a zoo. The isolates were clearly assigned to species level by biochemical identification systems, Fourier-transform infrared-spectroscopy, Matrix-assisted laser desorption/ionization-time of flight mass spectrometry and partial *rpoB* sequencing, respectively. All ten isolates turned out to represent the same sequence type, strongly indicating a cluster of infections by clonally-related isolates as could be demonstrated for the first time for this species using multilocus sequence typing. Unequivocal demonstration of high relatedness of the isolates could also be demonstrated by Fourier-transform infrared-spectroscopy. All isolates were lacking the diphtheria toxin encoding *tox*-gene, but were phospholipase D-positive.

**Conclusions:**

Our results indicate that water rats represent a suitable new host species that is prone to infection and must be regarded as a reservoir for potentially zoonotic *Corynebacterium ulcerans*. Furthermore, the applied methods demonstrated persistent infection as well as a very close relationship between all ten isolates.

## Background

The three *Corynebacterium* (*C*.) species *C. diphtheriae, C. ulcerans* and *C. pseudotuberculosis* form the *C. diphtheriae* group as recently shown by 16S rRNA gene sequence analysis and DNA-DNA hybridization studies [1-3]. Strains of this group might carry lysogenic β-corynephages which can harbor the *tox*-gene encoding diphtheria toxin (DT), a virulence factor inhibiting protein synthesis [4-6]. Moreover, both *C. ulcerans* and *C. pseudotuberculosis* may produce the dermonecrotic exotoxin phospholipase D, a major virulence factor involved in caseous lymphadenitis affecting mainly sheep, goats, and horses [4]. Recently, further putative virulence factors were identified in *C. ulcerans* including neuraminidase H, endoglycosidase E, subunits of adhesive pili and a gene coding for a putative ribosome-binding protein with striking structural similarity to Shiga toxins [7].

While *C. diphtheriae* carriage is nearly exclusively restricted to humans, toxigenic *C. ulcerans* are zoonotic pathogens and have been found in various animal species with contact to humans such as livestock as well as companion and laboratory animals including cows with mastitis [8-10], a goat with meningoencephalitis [11], an asymptomatic farm pig linked to a human case of diphtheria [12], macaques with mastitis or respiratory disease [13,14] or without symptoms [15], ferrets with infection derived from cephalic implants [16], pet dogs and cats displaying nasal discharge [17-22] and asymptomatic shelter dogs [17,23].

A number of studies have also outlined *C. ulcerans* isolations from wild, exotic and zoo animal species such as wild boars and a roe deer with abscess formation in Germany [24-26], Richardson ground squirrels with gangrenous dermatitis in Canada [27], two otters from UK [28], two killer whales and a lion from the same zoo in Japan [29] and a dromedary camel with purulent lymphadenitis [30]. Investigations on the toxicity with respect to DT were infrequently carried out, but some of the former isolates were toxigenic [12,15,20,22,31] or non-toxigenic *tox*-bearing (NTTB) strains [24-26], the latter being a phenotype originally described for *C. diphtheriae* strains [32] and recently also seen in *C. ulcerans* [33]. In recent years, diphtheria and diphtheria-like infections with toxigenic *C. ulcerans* have outnumbered those caused by toxigenic *C. diphtheriae* in many industrialized countries [33-35]. In contrast to *C. diphtheriae*-caused disease in humans, most human *C. ulcerans* diphtheria cases are associated with animal contact [33,35]. Since *C. ulcerans* has been increasingly isolated as an emerging zoonotic agent of diphtheria and other infections from different animal species, the aim of this study was the comprehensive characterization of ten *C. ulcerans* isolations from a yet undescribed host species, which caused significant morbidity within the same group of zoo animals.

## Results

### Gross pathology and histopathology

**Figure 1:**
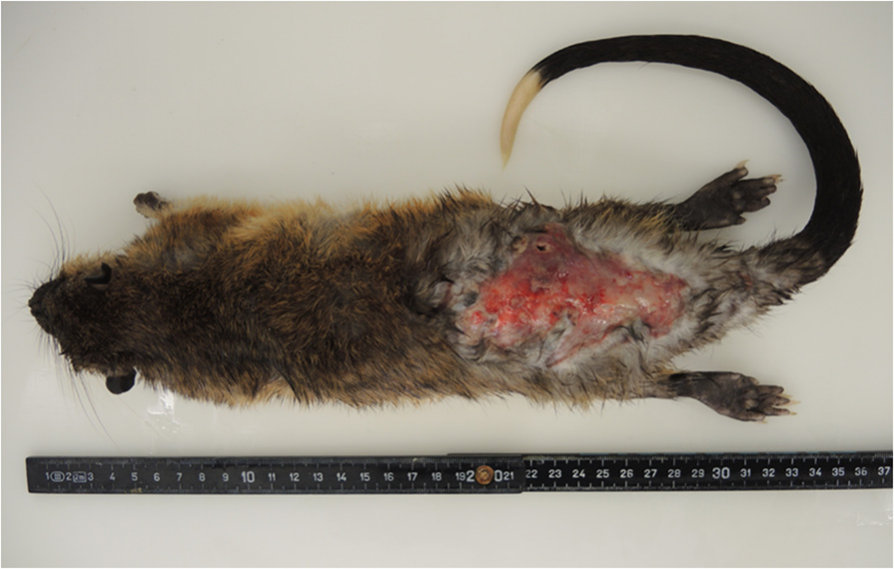
**Ulcer caused by a bite wound in a water rat from this study, concomitantly populated by** ***Corynebacterium ulcerans.***

Postmortem examinations were performed on cases no. I and VII. The adult male water rats were in poor body condition. Both animals showed multiple to coalescing deep cutaneous ulcers with irregular lateral margins in the caudal area of the dorsum measuring up to 3 × 10 cm (Figure 1).

Microscopic examination of the skin lesions of both rats revealed severe ulceration with extensive necrosuppurative dermatitis extending deeply into the subcutis and partially even underlying skeletal muscles with numerous intralesional colonies of bacilli as well as fragments of plant material and hair. Using Grocott methenamine silver (GMS) or Ziehl-Neelsen (ZN) stain or periodic acid Schiff (PAS) reaction neither fungal organisms nor acid fast bacilli could be detected.

### Bacterial isolates

In total ten isolates of *C. ulcerans* could be cultivated from eight water rats from the same group (Table 1). The bacteria grew after 24 hours on Columbia blood agar as whitish, chalk-like colonies, approximately 0.5-1.0 mm in diameter and mostly in moderate to high numbers from skin swabs, which were taken from infected ulcers. In animal no. I, *C. ulcerans* could be isolated from all organs in high numbers. The growth characteristics were consistent with coryneform bacteria [4]. No growth was observed on Gassner agar. After prolonged incubation for an additional 24 hours the irregular, dry colonies reached a size of 1–2 mm in diameter, surrounded by a narrow zone of beta-hemolysis. Gram staining revealed regular gram-positive coccobacilli. All isolates displayed the reverse CAMP phenomenon with *Staphylococcus aureus* and a regular CAMP reaction with *Rhodococcus equi*, thus indicating phospholipase D activity [4].

**Table 1:** **Origin of** ***Corynebacterium ulcerans*** **field isolates investigated in this study as well as gross pathology results from respective necropsies (1.0: male, 0.0.1: undetermined sex)**

**Case no.**	**Animal no.**	**Isolate ID**	**Year of isolation**	**Tissue with positive proof (isolate not stored)**	**Sex**	**Clinical presentation, gross pathology and histopathology**
1	I	131010012-1	2013	Skin at necropsy	1.0	Found dead with two 2 × 2 cm cutaneous ulcers on caudal dorsum and tail base; severe multifocal ulcerative necrosuppurative dermatitis with involvement of musculature; mild multifocal chronic lymphoplasmacytic tubulointerstitial nephritis; poor body condition; moderate postmortem changes
2	I	131010012-2	2013	Intestine at necropsy (liver, spleen, kidney, lung, intestinal lymph node)		
3	II	131011719	2013	Skin	1.0	Cutaneous ulcer on caudal dorsum
4	III	131013415-1	2013	Skin	0.0.1	Cutaneous ulcer on caudal dorsum
5	IV	131013415-2	2013	Skin	0.0.1	Cutaneous ulcer on caudal dorsum
6	V	131015432-10	2013	Skin	1.0	Cutaneous ulcer on caudal dorsum
7	VI	131015432-13	2013	Skin	1.0	Cutaneous ulcer 1 on caudal dorsum
8	VI	131015432-14	2013	Skin	1.0	Cutaneous ulcer 2 on caudal dorsum
9	VII	141001018	2014	Skin at necropsy	1.0	Multiple to coalescing cutaneous ulcers measuring up to 10 × 3 cm on caudal dorsum; multifocal ulcerative necrosuppurative dermatitis with involvement of musculature; focal chronic lymphoplasmacytic myocarditis; chronic lymphoplasmacytic tubulointerstitial nephritis; poor body condition; marked postmortem changes
10	VIII	141001548	2014	Skin	0.0.1	~2 × 2 cm cutaneous ulcer on caudal dorsum

### Biochemical studies

The conventional biochemical tests revealed corresponding results for catalase activity, esculin hydrolysis, urea hydrolysis and glucose acidification (all positive) as well as cytochrome oxidase, sucrose, maltose, D-xylose and D-mannitol acidification and nitrate reduction (all negative). Varying results (7 × positive, 3 × negative) were observed for D-trehalose according to Table 2. Using API Coryne or Vitek2-compact together with the ANC (for corynebacteria and anaerobes) and CBC (for coryneform bacteria) card all ten isolates were correctly identified as *C. ulcerans.* For details see Table 2.

**Table 2:** **Variable antimicrobial drug susceptibility testing by broth microdilution susceptibility testing with Merlin Micronaut system (μg/ml), biochemical characteristics, API Coryne and Vitek2-compact profiles of 10** ***Corynebacterium ulcerans*** **field isolates from water rats**

**isolate ID**	**TET**	**TILM**	**D-Trehalose**	**API Coryne profile;**	**Vitek2 CBC biotype number**	**Vitek2 ANC biotype number**
				**(interpretation/ % ID)**	**(interpretation/ % ID)**	**(interpretation/ % ID)**
131010012-1	R (>16)	R (32)	-	0 111 326 (Cul/99.7)	15431340407010 (Cul/97.0)	2363060410505 (Cul/99.0)
131010012-2	R (>16)	R (32)	+	0 111 326 (Cul/99.7)	15431340447010 (Cul/95.0)	2363020410505 (Cul/99.0)
131011719	R (>16)	R (32)	+	0 111 326 (Cul/99.7)	15031340406010 (Cul/99.0)	2363021414505 (Cul/91.0)
131013415-1	S (≤1)	R (32)	+	0 111 326 (Cul/99.7)	15431340447010 (Cul/94.0)	2363020400505 (Cul/97.0)
131013415-2	S (≤1)	R (32)	-	0 111 326 (Cul/99.7)	15030340407010 (Cul/97.0)	2363020410505 (Cul/99.0)
131015432-10	S (≤1)	R (32)	(+)	0 111 326 (Cul/99.7)	15431340447010 (Cul/94.0)	2363060410505 (Cul/95.0)
131015432-13	S (≤1)	I (16)	+	0 111 366 (Cul/99.9)	15431340447010 (Cul/94.0)	2363020410505 (Cul/99.0)
131015432-14	S (≤1)	I (16)	-	0 111 326 (Cul/99.7)	15431340407010 (Cul/97.0)	2363021410505 (Cul/95.0)
141001018	S (≤1)	I (16)	+	0 111 767 (Cul/99.9)	15431340407010 (Cul/98.0)	2363060412505 (Cul/90.0)
141001548	R (16)	I (16)	+	1 111 326 (Cul/98.0)	15431340407010 (Cul/97.0)	2363020410505 (Cul/99.0)

Susceptibility was determined by using the Clinical Laboratory Standards Institute criteria for broth microdilution susceptibility testing for *Corynebacterium* spp [50].

*TET* tetracycline,*TILM* tilmicosin, *R* resistant, *I* intermediate susceptible, *S* susceptible phenotype *+* positive, *−* negative, *(+)* weak reaction, *Cul* *C. ulcerans.*

### Antimicrobial susceptibility testing

All *C. ulcerans* isolates were *in vitro* susceptible (in brackets minimum inhibitory concentrations [MIC] in μg/ml) to amoxicillin/clavulanic acid (2/1), ampicillin (≤0.125-0.25), apramycin (≤8), cefquinome (≤1), ceftiofur (≤1), cephalothin (≤4), enrofloxacin (≤0.0625), erythromycin (≤0.125-0.25), florfenicol (≤1-2), gentamicin (≤2), neomycin (≤8), penicillin G (≤0.0625-0.125), trimethoprim/ sulfamethoxazol (0.5/9.5), and tiamulin (≤4-8). A resistant phenotype was recorded for clindamycin (4), colistin (≥4), and spectinomycin (64–128). Six and four isolates were susceptible (≤1) and resistant (16) to tetracycline, respectively and four and six isolates showed intermediate susceptibility (16) and resistance (16–32) to tilmicosin, respectively. For tests showing varying results see Table 2.

### Matrix-assisted laser desorption/ionization-time of flight mass spectrometry (MALDI-TOF MS)

Using MALDI-TOF mass spectrometry and the BioTyper database all ten isolates were identified to the species level as *C. ulcerans* with a score level between 2.0 and 2.2, using the direct smear method in sample preparation. Furthermore, identification of the concomitant bacterial flora comprising *Escherichia coli*, *Buttiauxella agrestis*, *Proteus* spp., *Aeromonas* spp., *Alcaligenes faecalis*, *Acinetobacter johnsonii*, *Streptococcus dysgalactiae*, *Enterococcus* spp., *Staphylococcus aureus* and *Clostridium perfringens* was also carried out by MALDI-TOF MS.

### Fourier Transformation-Infrared Spectroscopy (FT-IR)

**Figure 2:**
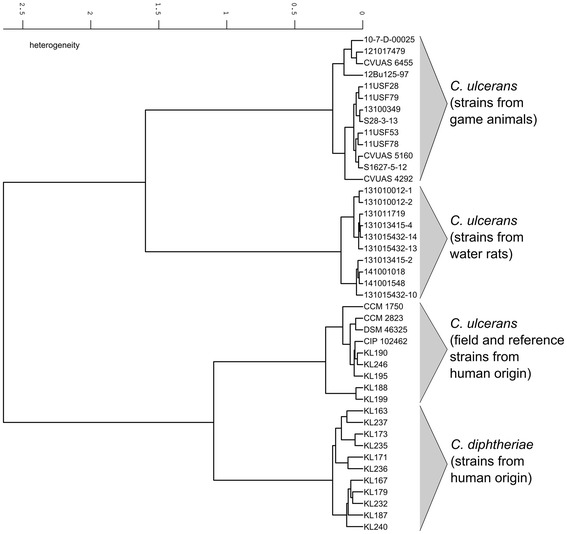
**Cluster analysis of respective spectra obtained by Fourier-transform infrared-spectroscopy (FT-IR) using OPUS Software (vers. 4.2, BrukerOptics).** In each case two IR-spectra of isolates from water rats and a selection of several *C. ulcerans* and *C. diphtheriae* strains were used for calculation with Ward’s algorithm. The dendrogram obtained depicts the arrangement of isolates in groups according to their spectral differences.

The comparison of the infrared (IR)-spectra of the ten isolates from water rats with a collection of field and reference strains showed a clear separation in two main branches for the closely related species *C. diphtheriae* and *C. ulcerans* (Figure 2). Inside the *C. ulcerans* branch all isolates from water rats cluster compactly together, closely adjacent to a group of spectra formed by the reference strains which were isolated from human sources. A group of *C. ulcerans* from game animals could also be distinguished by FT-IR [25] and clustered distant from the human and rat *C. ulcerans*.

### *tox* PCR

All ten isolates turned out to be non-toxigenic.

### *rpoB* analysis

**Figure 3:**
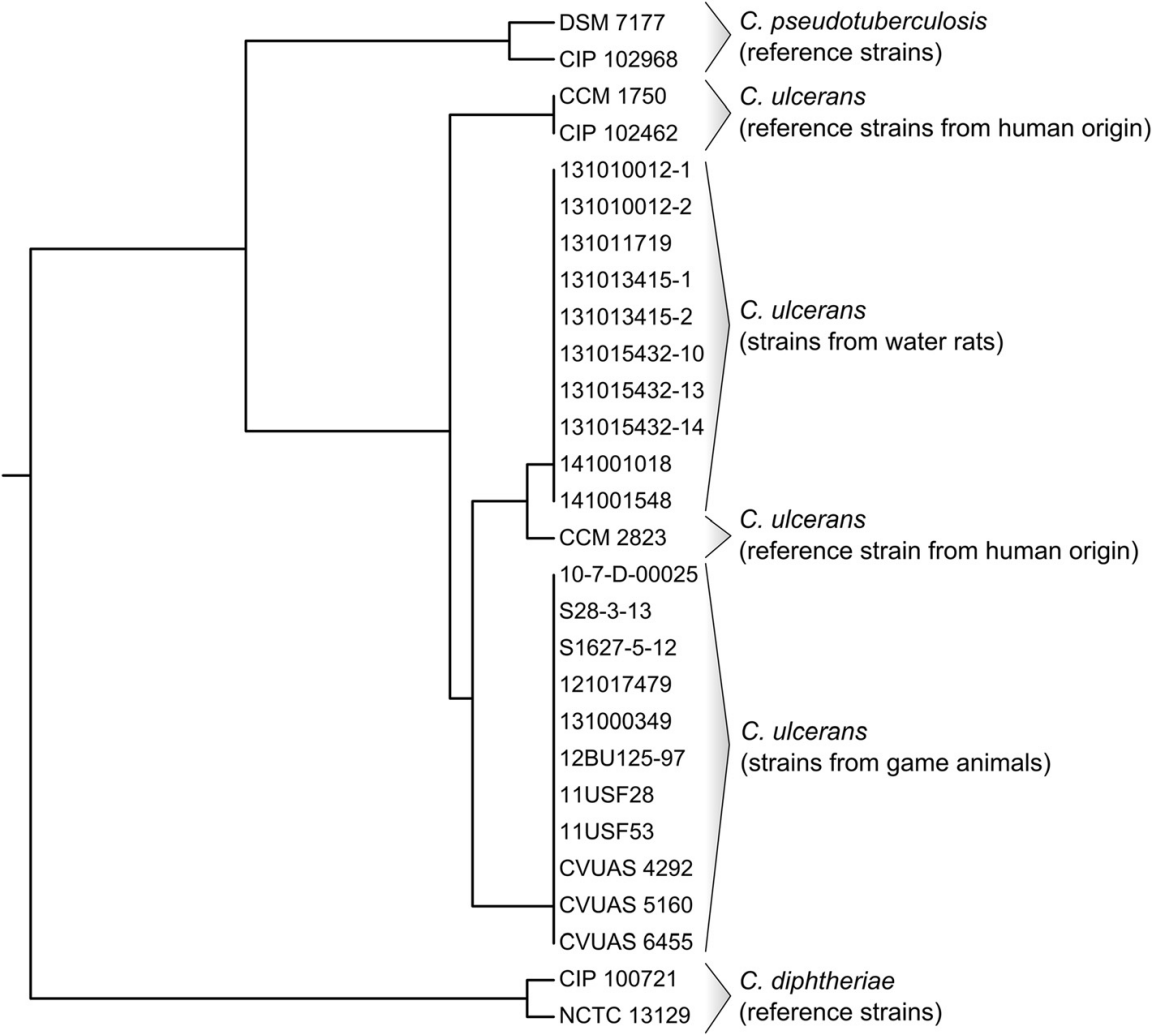
**Dendrogram of aligned partial** ***rpoB*** **sequences (406 bp) of** ***Corynebacterium ulcerans*** **isolates from water rats 131010012–1 (GenBank accession no. KM595079), 131010012–2 (KM595080), 131011719 (KM595081), 131013415–1 (KM595082), 131013415–2 (KM595083), 131015432–10 (KM595084), 131015432–13 (KM595085), 131015432–14 (KM595086), 141001018 (KM595087), and 141001548 (KM595088), compared to sequences from** ***C. ulcerans*** **strains isolated from game animals [** 25 **]** **; all sequences identical with GU818735) 10-7-D-00025, 121017479, CVUAS 6455, 12Bu125-97, 11USF28, 131000349, S28-3-13, 11USF53, CVUAS 5160, S1627-5-12, and CVUAS 4292, as well as humans CCM 1750 (GU818737), CIP 102462 (GU818739), CCM 2823 (GU818738),** ***C. pseudotuberculosis*** **DSM 7177 (GU818740), and CIP 102968 (AY492239) and** ***C. diphtheriae*** **strains CIP 100721 (AY492230), and NCTC 13129 (BX248355).**

Khamis et al. [36,37] have shown that the gene for the RNA polymerase beta subunit (*rpoB*) features a better degree of variability for the identification of *Corynebacterium* species than commonly used 16S rRNA sequences. Hence *rpoB* was analysed for all ten new isolates as the fragment was already sequenced during multilocus sequence typing (MLST) analysis. All isolates showed no difference in partial *rpoB* sequences [GenBank accession no. KM595079 - KM595088]. A *rpoB* sequence-based phylogenetic analysis of the isolates obtained from water rats as well as from humans and game animals is depicted in Figure 3. Isolates from water rats cluster together with DT-negative human strain CCM 2823 but distantly to all DT-negative strains from game animals and apart from human DT-positive strains CCM 1750 and CIP 102462.

### MLST analysis

**Figure 4:**
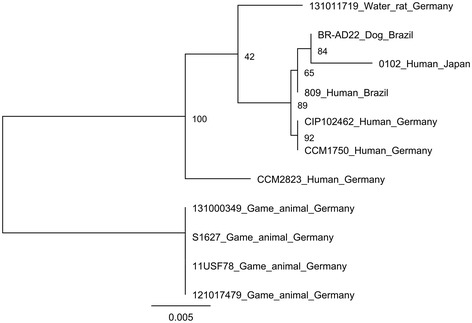
**Dendrogram of aligned concatenated housekeeping genes** ***atpA*** **,** ***dnaK*** **,** ***fusA*** **,** ***rpoB*** **, and** ***odhA*** **(1.813 bp) of** ***Corynebacterium ulcerans*** **isolates from a water rat compared to sequences from game animal and human strains.**

All isolates from water rats had the same allele type in six housekeeping genes. For *leuA* amplicons of only four isolates were obtained but these were also identical. None of the allele types observed corresponded to previously published types of *C. diphtheriae* strains (http://pubmlst.org/cdiphtheriae/). Two clusters were discernible in a phylogenetic tree based on concatenated sequences of five housekeeping genes of *C. ulcerans* strains of different sources (Figure 4). The rat isolates, represented by isolate 131011719, grouped together with human isolates and with an isolate from a dog, while the game animal isolates were clearly separated, forming their own cluster.

## Discussion

Within the genus *Corynebacterium* the *C. diphtheriae* group is the most relevant concerning pathogenicity and public health impact [24,38]. With respect to its zoonotic potential *C. ulcerans* is one of the most important members of the genus and was referred to as an emerging pathogen [7,34,39,40]. Numerous reports state zoonotic potential by contact with companion or farm animals [12,22,41], but proven transmission of a toxigenic *C. ulcerans* strain between an animal and a human by isolation of an identical toxigenic *C. ulcerans* strain from an animal and a human contact person is documented only in four reported cases involving two dogs [19,41], a cat [20] and a pig [12], respectively. During outbreaks in animal collections *C. ulcerans* isolates have so far never been subjected to detailed analysis, nor have they been tested for their relatedness. We have recently demonstrated a considerable homology between strains from game animals from different localities in Germany [25]. According to MLST data *C. diphtheriae* with its currently 335 sequence types (http://pubmlst.org/cdiphtheriae/) is a weakly clonal species, while the population structure of *C. ulcerans* is widely unknown. For this study an existing MLST scheme for *C. diphtheriae* [42] was adapted with good success, in that all seven allelic loci gave reasonable results also for *C. ulcerans*. A significant advantage of this method is that the data produced are portable, reproducible, and unambiguous compared to other methods for strain discrimination (e.g. ribotyping) [42]. The MLST data indicate a very close relationship of the isolates suggesting spread of a clonally-related lineage. The question of origin of this isolate, whether it originated from animals in close proximity or whether it has been introduced by zookeepers of the zoological garden requires further investigations. A phylogenetic tree based on housekeeping genes obtained from available whole genomes places the rat isolates in closer proximity to human than to game animal isolates. On the other hand, the assessment of *rpoB* sequences reveals major grouping effects between DT-negative and DT-positive *C. ulcerans* strains in particular (Figures 3 and 4). Whether this reflects a common phylogeny of *C. ulcerans* strains from humans and water rats needs to be further determined using a broader collection of isolates.

Concerning rodent host species and clinical symptoms this report bears remarkable analogies to an outbreak among 350 squirrels from Canada which were captured within the city of Calgary; 63 of them developed dermatitis, conjunctivitis, pharyngitis or septicemia, respectively, when held together in cages within a research facility [27]. As in the case presented here, cutaneous lesions were associated with bite injuries transmitted by asymptomatically infected carrier cage mates and were most likely caused by seasonal group hierarchical fighting. Unfortunately, no toxicity testing was performed on the outbreak strain from the squirrels. *C. ulcerans* was also isolated from bite injuries, abscesses and pneumonic lung tissue in primates and injection of these isolates led to deep ulcers and necrotic abscesses in guinea pigs as another rodent species [43]. In humans, reported cases of cutaneous diphtheria by toxigenic *C. ulcerans* or *C. diphtheriae* by far outnumber those rare cases of skin infections through non-toxigenic *C. ulcerans* strains [21,44-47], nevertheless, they do occur [21]. There is no information available concerning the zoonotic potential of *C. ulcerans* isolated from water rats. All ten isolates in our study turned out to be non-toxigenic. Recent studies have shown additional virulence factors in *C. ulcerans* besides DT and phospholipase D [7]. Human diphtheria can also be attributed to the emergence of non-toxigenic strains causing atypical disease [42]. Strains, which produce only phospholipase D but not DT are also capable to cause severe systemic disease in humans, such as pneumonia and granulomatous nodules in the respiratory tract [23]. All isolates from this study were highly similar with respect to their antimicrobial pattern and showed at least partial resistance to clindamycin, colistin, spectinomycin, tetracycline and tilmicosin *in vitro*. Albeit clindamycin was successfully used to treat animal infection [21], resistance to this antimicrobial substance was noted for several human strains of *C. ulcerans* which were in some cases also resistant to erythromycin [34] and levofloxacin [17]. One report states oxacillin-resistance in an isolate, which also proved to be clindamycin sensitive [30]. Four of the here investigated ten isolates were resistant to tetracycline.

As also shown in previous studies, biochemical differentiation between *C. ulcerans* and *C. pseudotuberculosis* can be problematic [33,36,37,48]. Basic conventional tests might be less discriminating between the two species [4]. Furthermore, they are generally difficult to standardize, thus they may lead to varying and less comparable results. By use of the standardized systems API Coryne and Vitek2-compact all ten isolates from water rats could be correctly identified as *C. ulcerans*. We have shown recently, that these commercial biochemical test systems were insufficient for the unequivocal identification of *C. ulcerans* isolates from game animals [25]. In contrast, MALDI-TOF MS has been recently proven to differentiate both, potentially toxigenic *Corynebacterium* species and coryneform bacteria [48,49], and this was also true for this study. In addition, we demonstrate that FT-IR can also clearly identify *C. ulcerans* at species level, showing a high degree of spectral relatedness of the isolates under study. Since partial *rpoB* sequencing is more discriminatory when compared to 16S rDNA sequencing, a cutoff value of ≤95% similarity proved suitable for species identification within corynebacteria [37] and also clearly enabled species identification in the here described isolates. As suggested from earlier studies [24-26] comparison of partial *rpoB* gene sequences confirmed a close relationship of all ten isolates in this study, since no variations in these sequences were found.

## Conclusion

We have documented ten *C. ulcerans* isolates from a yet unknown rodent host species. For correct understanding of epidemiology and to unequivocally determine the involved pathogen correctly to species level, commercial biochemical tests and additional approved methods like FT-IR, MALDI-TOF MS and *rpoB* sequencing were sufficient to prove the *C. ulcerans* infection. Furthermore, the latter methods and also MLST demonstrated a very close relationship between all ten isolates. Future studies should include further *C. ulcerans* isolates from wildlife in virulence profiling and in phylogenetic typing to fully understand their properties and possible zoonotic consequences.

## Methods

### Case description

*Hydromys chrysogaster*, commonly known as “rakali” or “golden bellied water rats”, is a rodent species native to Australia. A breeding group in a zoo was housed in an enclosure of 15.2 m^2^ (29 m^3^) with a water basin of 4 m^3^ and several hiding places. During the mating season in June 2013, male water rats displayed numerous skin ulcera, which were associated with bite wounds from intra-species aggression (Figure 1). Mostly subadult males or animals representing individuals with a low level in the group’s hierarchy were affected. After sampling skin ulcera were successfully treated by local oxytetracycline aerosol application. In severe cases, affected animals were found dead or so deeply moribund that euthanasia was considered the only option and necropsy was performed. Euthanasia was performed according to 2013 edition of AVMA guidelines for the euthanasia of animals (https://www.avma.org/KB/Policies/Documents/euthanasia.pdf). Briefly, pentobarbital-sodium 300 mg/ml (Release, Wirtschaftsgenossenschaft deutscher Tierärzte eG, Garbsen, Germany) was administered intraperitoneally. Animal husbandry fulfilled ethical standard guidelines according to the code of ethics and animal welfare of the world association of zoos and aquariums (WAZA; http://www.waza.org/files/webcontent/1.public_site/5.conservation/code_of_ethics_and_animal_welfare/Code%20of%20Ethics_EN.pdf). We further declare that the present study complies with national guidelines. According to the Hesse State Council (Giessen, Germany) the animal work does not require formal approval by its ethics committee or general approval with respect to German law.

### Bacteria isolation

Isolates of *C. ulcerans* were obtained during routine bacteriological investigations following skin swabbing (case no. 3–8, 10; Table 1) or post mortem examinations (animal no. I, VII; Table 1) from moribund and dead water rats between July 2013 and February 2014. Following full gross examination, tissue specimens of skin, mesenteric lymph node, lung, liver, kidney, intestine, brain, and conspicuous lesions were fixed in 10% neutral buffered formalin and embedded in paraffin. Sections (3 μm) were cut and routinely stained with haematoxylin and eosin. Additionally, sections of skin were stained with GMS, PAS and ZN. Native tissue samples were processed for bacterial culture. Briefly, organ samples and marginal areas of abscesses were flame sterilized and the surface of a fresh cut was directly inoculated onto culture media. Agar plates were incubated for up to 48 hours at 37°C using aerobic (Columbia agar with 5% sheep blood and Gassner agar; all Oxoid, Wesel, Germany), capnophilic and anaerobic conditions (Schaedler; Oxoid), respectively.

### Phenotypic characterization

Phenotypic characterization was performed by standard microbiological procedures: Haemolytic properties of the bacteria were examined on blood agar containing 5% sheep blood, microscopic examinations of fixed smears were performed using Gram staining. Bacterial colonies were tested for catalase activity with 3% H_2_O_2_ on microscopic slides. Isolates of coryneform bacteria were subjected to conventional biochemical tests according to Funke et al. and Hommez et al. [4,10], including nitrate reduction, urea hydrolysis, esculin hydrolysis, acid production from glucose, sucrose, maltose, D-trehalose, D-xylose and D-mannitol (all substrates Merck, Darmstadt, Germany). The tests were evaluated after prolonged incubation at 37°C on days 2, 7, and 14. For further characterization standardized test systems were used, i.e. API Coryne and Vitek2-compact, the latter with respective card systems for both CBC and ANC (all bioMérieux, Nürtingen, Germany). All tests were performed according to the manufacturer’s instructions. Since *C. ulcerans* and *C. pseudotuberculosis* both display a reverse CAMP phenomenon when tested with an orthogonal growing *Staphylococcus aureus* American Type Culture Collection (ATCC [Manassas, VA, USA]) 25923 and a regular CAMP reaction with *Rhodococcus equi* ATCC 33701 both tests were routinely carried out.

*C. diphtheriae* KL163, KL167, KL171, KL173, KL179, KL187, KL232, KL235-237, KL240 and *C. ulcerans* KL188, KL190, KL195, KL199, KL246 isolates from humans (National Consiliary Laboratory on Diphtheria)*, C. ulcerans* CCM 1750 and CCM 2823 (Czech Collection of Microorganisms, Masaryk University, Brno, Czech Republic), *C. ulcerans* CIP 102462 (Institute Pasteur, Paris, France), *C. ulcerans* DSM 46325^T^ (DSMZ - German Collection of Microorganisms and Cell Cultures, Braunschweig, Germany), *C. ulcerans* from wild animals [25] and *C. pseudotuberculosis* DSM 7177, CIP 102968 were used as reference strains for comparison.

### Antimicrobial susceptibility testing

Antimicrobial drug susceptibility testing was carried out using the broth microdilution susceptibility testing with a commercial system (Merlin Micronaut, Bornheim, Germany) according to the instructions of the manufacturer. The test design (Merlin according to AVID [Arbeitskreis veterinärmedizinische Infektionsdiagnostik of the German Veterinary Society] guidelines) contained the following 19 antimicrobial substances for minimum inhibitory concentration (MIC) testing: amoxicillin/clavulanic acid, ampicillin, apramycin, cefquinome, ceftiofur, clindamycin, colistin, cephalothin, enrofloxacin, erythromycin, florfenicol, gentamicin, neomycin, penicillin G, spectinomycin, trimethoprim/ sulfamethoxazol, tetracycline, tiamulin, and tilmicosin. Susceptibility was determined by assessing clinical breakpoints according to Clinical Laboratory Standards Institute criteria for broth microdilution susceptibility testing for *Corynebacterium* spp. [50].

### Identification by MALDI-TOF MS

Potential coryneform isolates were selected from the culture plates and then subjected to steel-targets according to manufacturer’s instructions (BrukerBiotyper, BrukerDaltonics, Bremen, Germany). Isolates were prepared using the direct smear method and analyzed by MALDI-TOF MS using Biotyper Version V3.3.1.0. The database used (DB 4613, BrukerDaltonics) comprised spectra from 71 *Corynebacterium* species including *C. diphtheriae*, *C. ulcerans*, and *C. pseudotuberculosis*.

### Cluster analysis of infrared spectra of isolates obtained by FT-IR

All bacterial isolates were cultivated independently in 5–7 replicates at 37°C for 24 h on sheep blood agar plates (Oxoid). Harvesting of cells, preparation of bacteria films on zinc selenide plates, drying and handling were performed as described previously [51]. The dried bacteria films were used directly for examination by FT-IR spectroscopy. Infrared spectra were recorded for each sample in a transmission mode from 500 to 4000 cm^−1^ with an FT-IR spectrometer (Tensor27 with HTS-XT-module, BrukerOptics, Ettlingen, Germany). Acquisition and first analysis of data were carried out using OPUS Software (vers. 4.2, BrukerOptics). IR spectra of isolates from water rats and a selection of several *C. ulcerans* and *C. diphtheriae* strains were compared by cluster analysis (cf. [52,53]). For cluster analysis, the second derivation of the vector normalized spectra in the wave number range of 500–1400 cm^−1^ and 2800–3000 cm^−1^ were used for calculation with Ward’s algorithm (OPUS 4.2; [54]). The dendrogram obtained depicts the arrangement of isolates in groups according to their spectral differences (Figure 2).

### *tox* PCR

The *tox* gene amplification was assessed by using primers DT1 and DT2 [55] in a modified PCR-protocol described previously [24].

### MLST

MLST was performed according to Bolt et al. [42]. Briefly, seven housekeeping genes *atpA, dnaE, dnaK, fusA, leuA, odha,* and *rpoB* were amplified and both strands of the PCR products were sequenced by Eurofins MWG (Ebersberg, Germany) using the amplification primers. Primer pairs for *leuA* (leuAulcf CGTTCACTTCTACAATTC and leuAulcr GCCGTGGTCAGTTTTCAT) and *dnaK* (dnaKulcf ACTTGGGTGGCGGAACCT and dnaKulcr TGGTAAAGGTCTCAGAA) were modified to improve amplification of *C. ulcerans leuA* and *dnaK* genes. Sequence analysis was done with Lasergene (DNASTAR, Madison, USA). Partial MLST was further performed for seven *C. ulcerans* strains from game animals [25] and from humans [20], which were also included in FT-IR analysis. Additional housekeeping gene sequences were obtained from available whole genome sequences of *C. ulcerans* human isolates 809 [7] [Accession-No. CP0022790] and 0102 [56] [AP012284] and of canine isolate BR-AD22 [7] [CP002791]. For calculating a phylogenetic tree of the concatenated sequences of *atpA*, *dnaK*, *fusA*, *rpoB*, and *odhA* genes we used RAxML (Randomized Axelerated Maximum Likelihood) 8 [57]. This is a program for sequential and parallel Maximum Likelihood based inference of large phylogenetic trees. In detail, we used the General Time Reversible model of nucleotide substitution under the Gamma model or rate heterogeneity. A total number of 100 bootstrap replicates with random seed were calculated and visualization of the tree was performed with Dendroscope 3 [58].

## Abbreviations

*C.*: *Corynebacterium*
MALDI-TOF MS: Matrix-assisted laser desorption/ionization-time of flight mass spectrometry
FT-IR: Fourier-transform infrared-spectroscopy
DT: Diphtheria toxin
MLST: multilocus sequence typing
NTTB: non-toxigenic *tox*-bearing
GMS: Grocott methenamine silver
ZN: Ziehl-Neelsen
PAS: Periodic acid schiff
ANC: Corynebacteria and anaerobes
CBC: Coryneform bacteria
MIC: Minimum inhibitory concentration
IR: Infrared
CCM: Czech collection of microorganisms
CIP: Collection of institute pasteur
ATCC: American type culture collection
KL: Strain collection of the national consiliary laboratory on diphtheria
DSM: German collection of microorganisms and cell cultures
AVID: Arbeitskreis veterinärmedizinische Infektionsdiagnostik
